# Perspectives on nonprescription antibiotic use among Hispanic patients in the Houston metroplex: A qualitative study

**DOI:** 10.1017/ash.2023.237

**Published:** 2023-09-29

**Authors:** Lindsey Laytner, Patricia Chen, Susan Nash, Michael Paasche-Orlow, Kiara Olmeda, Juanita Salinas, Richard Street, Roger Zoorob, Barbara Trautner, Larissa Grigoryan

## Abstract

**Background:** Nonprescription antibiotic use includes taking an antibiotic without medical guidance (eg, leftover antibiotics, antibiotics from friends or relatives, or antibiotics purchased without a prescription). Nonprescription use contributes to antimicrobial resistance, adverse drug reactions, interactions, and superinfections such as *Clostridioides difficile* colitis. Qualitative studies exploring perspectives regarding nonprescription use among Hispanic patients are lacking. We used the Kilbourne Framework for Advancing Health Disparities Research to identify factors influencing Hispanic patients’ nonprescription use and to organize our findings. **Methods:** Our study includes Hispanic primary-care clinic patients with different types of health coverage in the Houston metroplex who endorsed nonprescription use in a previous quantitative survey. Semistructured interviews explored the factors promoting nonprescription use in Hispanic adults. Interviews were conducted remotely, in English or Spanish, between May 2020 and October 2021. We used inductive coding and thematic analysis to identify the factors and motives for nonprescription use. **Results:** Of the 35 Hispanic participants surveyed, 69% were female and between the ages of 27 and 66. All participants had some form of healthcare coverage (eg, Medicare or private insurance, Medicaid, or the county financial assistance program). Participants reported obtaining antibiotics from their own leftover prescriptions and through trusted persons (eg, herbalists, pharmacists, friends/relatives, and others), buying them under the counter in US markets, and purchasing them without a prescription outside the United States. Thematic analysis revealed the factors contributing to nonprescription use (Fig. 1). Themes included beliefs that the ‘doctor visit was unnecessary,’ ‘limited direct access to healthcare’ in the United States (due to limited insurance coverage, high costs of the doctor’s visits and medications, and long clinic wait times), ‘more open indirect access to healthcare’ abroad and under the counter in the United States, and communication difficulties (eg, language barriers with clinicians, perceived staff rudeness, and gaps in health literacy). Figure 2 shows representative quotes across thematic domains. Participants expressed having confidence in medical recommendations from pharmacists and trusted community members in their social networks. **Conclusions:** Antibiotic stewardship interventions that include pharmacist-driven patient education regarding appropriate antibiotic use may decrease nonprescription antibiotic use in Hispanic communities. Additionally, improving access to care while addressing communication barriers and cultural competency in clinics may improve primary care delivery and reduce potentially unsafe antibiotic use.

**Figure:**
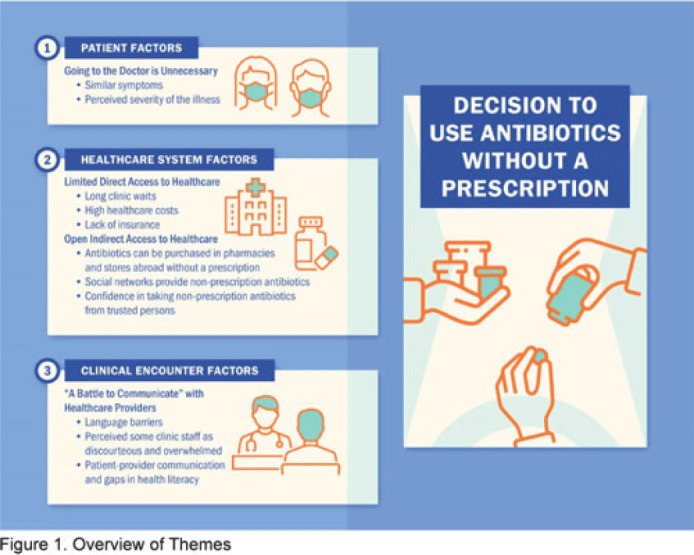

**Figure:**
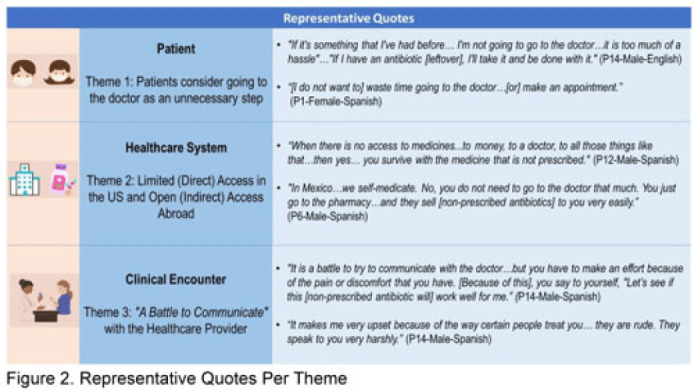

**Disclosure:** None

